# Effect of CRTH2 antagonism on the response to experimental rhinovirus infection in asthma: a pilot randomised controlled trial

**DOI:** 10.1136/thoraxjnl-2021-217429

**Published:** 2021-10-29

**Authors:** Hugo Farne, Nicholas Glanville, Nicholas Johnson, Tata Kebadze, Julia Aniscenko, Eteri Regis, Jie Zhu, Maria-Belen Trujillo-Torralbo, Onn Min Kon, Patrick Mallia, A Toby Prevost, Michael R Edwards, Sebastian L Johnston, Aran Singanayagam, David J Jackson

**Affiliations:** 1 National Heart and Lung Institute, Imperial College London, London, UK; 2 Imperial Clinical Trials Unit, Imperial College London, London, UK; 3 Guy’s Severe Asthma Centre, Guy’s and St Thomas’ NHS Foundation Trust, London, UK; 4 MRC & Asthma UK Centre in Allergic Mechanisms of Asthma, King’s College London, London, UK

**Keywords:** asthma, viral infection

## Abstract

**Background and aims:**

The chemoattractant receptor-homologous molecule expressed on T helper type 2 cells (CRTH2) antagonist timapiprant improved lung function and asthma control in a phase 2 study, with evidence suggesting reduced exacerbations. We aimed to assess whether timapiprant attenuated or prevented asthma exacerbations induced by experimental rhinovirus (RV) infection. We furthermore hypothesised that timapiprant would dampen RV-induced type 2 inflammation and consequently improve antiviral immune responses.

**Methods:**

Atopic patients with partially controlled asthma on maintenance inhaled corticosteroids were randomised to timapiprant (n=22) or placebo (n=22) and challenged with RV-A16 3 weeks later. The primary endpoint was the cumulative lower respiratory symptom score over the 14 days post infection. Upper respiratory symptoms, spirometry, airway hyperresponsiveness, exhaled nitric oxide, RV-A16 virus load and soluble mediators in upper and lower airways samples, and CRTH2 staining in bronchial biopsies were additionally assessed before and during RV-A16 infection.

**Results:**

Six subjects discontinued the study and eight were not infected; outcomes were assessed in 16 timapiprant-treated and 14 placebo-treated, successfully infected subjects. There were no differences between treatment groups in clinical exacerbation severity including cumulative lower respiratory symptom score day 0–14 (difference 3.0 (95% CI −29.0 to 17.0), p=0.78), virus load, antiviral immune responses, or RV-A16-induced airway inflammation other than in the bronchial biopsies, where CRTH2 staining was increased during RV-A16 infection in the placebo-treated but not the timapiprant-treated group. Timapiprant had a favourable safety profile, with no deaths, serious adverse events or drug-related withdrawals.

**Conclusion:**

Timapiprant treatment had little impact on the clinicopathological changes induced by RV-A16 infection in partially controlled asthma.

**Key messages**

**What is the key question?**

- Does the chemoattractant receptor-homologous molecule expressed on T helper type 2 cells (CRTH2) antagonist timapiprant attenuate or prevent asthma exacerbations induced by experimental rhinovirus infection?

**What is the bottom line?**

- In this single-centre, double-blind, placebo-controlled, parallel group randomised controlled study, timapiprant treatment had minimal impact on the clinicopathological changes induced by experimental rhinovirus infection in partially controlled asthma.

**Why read on?**

- There has been considerable interest in oral CRTH2 antagonists as treatments for asthma; our data suggest that these therapies do not improve clinical or immunological markers in asthma exacerbations.

## Introduction

Asthma is one of the most common chronic diseases affecting 240–300 million people worldwide with ~49 million deaths in 2017.^1^ Most of the morbidity, mortality and healthcare costs associated with asthma are due to acute increases in symptomatology called exacerbations, episodes commonly triggered by respiratory viruses, particularly rhinoviruses (RVs).^2^ Despite treatment, almost half of patients with asthma continue to experience exacerbations.^3^ These are often associated with an acute rise in ‘type 2’ inflammation, as demonstrated by increases in the type 2 cytokines interleukin (IL)−4, IL-5 and IL-13 in airways samples,^4 5^ which correlate with symptom severity.^5^ Type 2 inflammation has been shown to impair antiviral immunity,^6–8^ thus suppressing type 2 inflammation may confer beneficial effects on host immunity and exacerbation susceptibility. Recent clinical trial and real-world data confirm that targeting type 2 inflammatory pathways is effective in reducing asthma exacerbations.^9–13^

The chemoattractant receptor-homologous molecule expressed on T helper type 2 (Th2) cells (CRTH2) is a target for tackling type 2 inflammation. It is expressed on immune cells instrumental in promoting type 2 inflammation: eosinophils, basophils, Th2 cells and type 2 innate lymphoid cells (ILC2s). When bound to its endogenous ligand prostaglandin D_2_ (PGD_2_), CRTH2 receptor signalling leads to migration of Th2 cells, ILC2s, eosinophils and basophils,^14 15^ type 2 cytokine release by Th2 cells^16^ and ILC2s,^17^ eosinophil shape change and degranulation,^18^ and enhancement of IgE-mediated basophil degranulation.^19^ Thus, CRTH2 may be a central regulator of type 2 inflammation and represents a biologically plausible therapeutic target. Evidence for a role in asthma is supported by lower airways samples from patients with asthma showing increased levels of PGD_2_ and CRTH2 mRNA,^20 21^ increased expression of enzymes required for PGD_2_ synthesis^20 22^ and higher numbers of PGD_2_-responsive CRTH2^+^ cells^20 23 24^ compared with healthy controls. PGD_2_ has additionally been implicated in asthma exacerbations: PGD_2_ is increased during RV-induced exacerbations with levels correlating with exacerbation severity,^25^ by a variety of respiratory viruses in animal models of allergic asthma,^26^ and by bronchial allergen challenge in atopic individuals^27^ especially if they have asthma.^28^ Moreover, BAL PGD_2_ and CRTH2 mRNA are higher in patients with a recent asthma exacerbation.^20^ Clinical trials have included a phase 2a study of the CRTH2 antagonist timapiprant in asthma that observed a lower rate of exacerbations (3.8% in the pooled timapiprant dose groups vs 7.7% placebo; p=0.107), not statistically significant in the context of the study not being powered for this outcome.^29^ A recent publication of two phase 3 clinical trials in subjects with severe asthma found ‘consistent and modest reductions in exacerbation rates in both studies’, around 22%, although this failed to reach statistical significance.^30^ Collectively these data provide a rationale for the investigation of drugs targeting PGD_2_-CRTH2 signalling in asthma.

We have previously reported a model of experimental RV-induced exacerbation of asthma that provides a controlled experimental system to study the effects of novel therapies.^5 31^ The current trial was designed to assess the potential for the CRTH2 antagonist timapiprant to prevent or attenuate asthma exacerbations using this model. We hypothesised that timapiprant would attenuate type 2 inflammation and thus prevent RV-induced accentuation of clinical asthma severity including lower respiratory symptom scores, lung function decline and airway hyper-responsiveness.

## Methods

### Study design

This was a single-site, randomised, double-blind, parallel group, placebo-controlled trial examining the effect of the CRTH2 antagonist timapiprant on the response to RV infection in asthma.

### Participants

Non-smoking atopic subjects with partially controlled asthma maintained on inhaled corticosteroids (ICS) were eligible for the study. Subjects were aged 18–55 years with absent antibodies to RV-A16 in a microneutralisation assay^32^ at screening. Subjects were required to have a doctor diagnosis of asthma treated with ICS, airway hyper-responsiveness or a bronchodilator response of ≥12%, a positive skin prick test to any of a panel of aeroallergens,^31^ and an Asthma Control Questionnaire (ACQ)−6 score of >0.75. See online supplemental material for full inclusion and exclusion criteria. Written informed consent was obtained prior to participation.

10.1136/thoraxjnl-2021-217429.supp1 Supplementary data

### Randomisation and masking

Eligible subjects were randomly assigned to either one time a day timapiprant (50 mg) or matched placebo in a 1:1 ratio, according to a randomisation list generated by a statistician working independently of the trial that was encoded in the study database and concealed up to allocation. Subjects were randomised in blocks of four to ensure balanced allocation to each group. Subjects and study investigators were blinded to treatment allocation from randomisation until database lock. Treatment identity was concealed by using placebo identical in packaging, labelling, schedule of administration, appearance, taste and odour to timapiprant.

### Procedures

**Figure 1:**
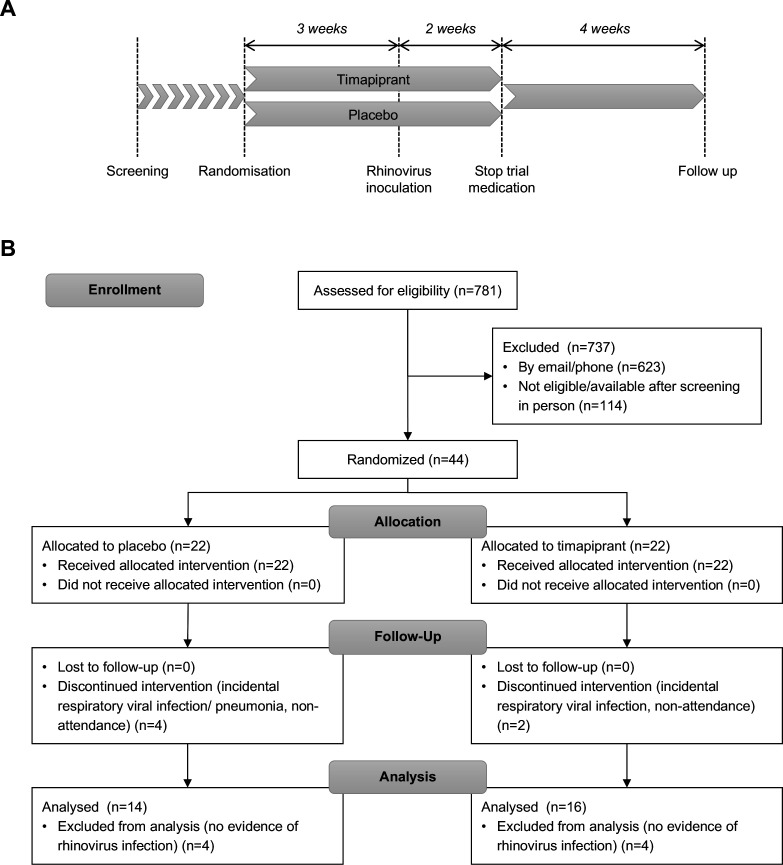
Study design and trial profile. (A) Schematic of study design. (B) Consolidated Standards of Reporting Trials flow diagram.

The study comprised a 3-week preinfection treatment phase after which both groups were inoculated with RV-A16, with treatment continuing until 2 weeks after inoculation, and a single convalescence visit 4 weeks later (figure 1A). Subjects completed daily diary cards of upper and lower respiratory symptoms (details in online supplemental material), home spirometry readings and medication use from randomisation until the end of the study. Subjects additionally attended for 11 study visits: baseline visits at randomisation (day −21) and after 13 days of treatment (day −8); an inoculation visit (day 0) when subjects received 100 tissue culture infective dose 50% of RV-A16 using methods as previously described^5^; and visits on days 2, 3, 4, 5, 7, 10 and 42 post inoculation. Clinical assessment and sampling at study visits included ACQ-6 score, clinic spirometry, bronchoprovocation challenge (to calculate the histamine provocation concentration producing a 20% fall in forced expiratory volume in 1s (FEV_1_) (PC_20_), exhaled nitric oxide (Fe_NO_), nasal lavage, nasal and bronchial lining fluid sampling using synthetic absorptive matrices (nasosorption and bronchosorption, respectively), and bronchial biopsies, with methods as per previous studies.^5 31^ See online supplemental material for further details.

RV-A16 infection was confirmed by positive qPCR for RV-A16 in nasal lavage at any time after inoculation or seroconversion (positive antibodies to RV-A16 at a titre of at least 1:4 at the final study visit). Virus copies were quantified in nasal lavage, bronchoalveolar lavage and sputum samples by qPCR as previously described.^5^

Concentrations of soluble mediators were quantified in the nasosorption and bronchosorption strip eluates as previously described.^5 25^ CRTH2 immunostaining was performed using rabbit anti-CRHT2 (abcam, UK) at a dilution of 1:300 and the EnVision peroxidase staining method (Agilent Dako, USA), method and quantification as previously described.^33^

### Outcomes

The primary endpoint was the total lower respiratory symptom score (sum of daily lower respiratory symptoms on days 0–14 after RV inoculation). This was chosen as it is well established that experimental RV infection in subjects with asthma induces lower respiratory symptoms consistent with mild-to-moderate exacerbations,^34^ whereas it very rarely results in a severe exacerbation requiring oral corticosteroids (the primary outcome for most phase 3 clinical trials). Secondary efficacy endpoints included upper respiratory symptoms, ACQ-6 scores, lung function (FEV_1_ and peak expiratory flow, PEF), PC_20_, Fe_NO_ and virus load. Exploratory outcomes included soluble mediator concentrations in the upper and lower airways, and CRTH2^+^ cells in immunohistochemistry-stained bronchial biopsies.

### Statistical analysis

An initial sample size calculation based on a previously completed study with similar design conducted at the same site^5^ indicated that 10 subjects per treatment group with confirmed RV infection were required, provided they had an ACQ-6 score of ≥1.5. Following difficulties in recruitment and improvements in ACQ-6 scores between screening and randomisation in several subjects, attributed to better compliance with maintenance asthma treatment on involvement in a clinical trial, the ACQ-6 cut-off was relaxed to >0.75. A revised sample size calculation, assuming a difference in mean score of 22.21 in the primary endpoint with a SD of 21.15, yielded 15 subjects per treatment group at 80% power using a two-sided test at the 5% significance level. This was grossed up for RV infection success (80%) and anticipated drop-out rate (15%) to yield 22 patients per treatment group.

Data were analysed using SAS V.9.4 (SAS Institute) and GraphPad Prism V.8 (GraphPad Software, USA). Continuous variables are presented as means and SD where data were consistent with a normal distribution, otherwise as medians and IQR. Correspondingly, the unpaired t-test and Mann-Whitney U test were used to analyse between groups. Categorical variables are presented as frequencies and percentages and analysed between groups using the χ^2^ test or Fisher’s exact test as appropriate. Differences between time points within groups were investigated by paired t tests for parametric or Wilcoxon’s signed-rank test for non-parametric data. Correlations were examined using Pearson’s and Spearman’s correlation tests depending on normality of the data. Differences were considered statistically significant at p<0.05. All p values are two sided.

## Results

Between 9 March 2016 and 22 November 2017, 44 patients were randomly assigned to timapiprant 50 mg (n=22) or placebo (n=22) (figure 1B). The mean (SD) ACQ-6 score improved for the overall cohort of 44 subjects from 1.54 (0.62) at screening to 1.31 (0.67) at randomisation (day −21), p=0.012. Six were withdrawn following randomisation prior to RV inoculation; four with incidental respiratory tract infections, two due to non-compliance with the study protocol (both did not attend a study visit or respond to contact in the following days). A further eight subjects failed to develop RV infections and were excluded from the analysis. In total, 30 subjects with confirmed infection completed the study and formed the analysis set, meeting the sample size calculation target of 30 subjects.

Subjects with confirmed infection were young (mean age 25.3 (6.9) years) with partially controlled asthma with a mean ACQ-6 score of 1.27 (0.75) and modest airflow obstruction (median FEV_1_ 93.5% (80.0%–100.0%) predicted) at randomisation (day −21). They had evidence of ongoing type 2 inflammation at baseline with a median Fe_NO_ of 34 (25–66) ppb and blood eosinophil count of 0.3 (0.2–0.4)×10^9^/L, suggesting they would be susceptible to RV-induced changes in asthma control and that biological pathways relevant to drug treatment were active. The baseline characteristics of subjects who were successfully infected were similar across treatment groups (table 1).

**Table 1:** Subject demographics and clinical characteristics at enrolment (day −21)

	Placebo (n=14)	Timapiprant (n=16)
Age (years)	25.4 (3.8)	25.3 (8.9)
Sex		
Male	5 (36%)	7 (44%)
Female	9 (64%)	9 (56%)
Body mass index (kg/m^2^)	23.8 (2.4)	24.8 (4.3)
Inhaled corticosteroid dose (beclometasone dipropionate equivalent μg/day)	366 (356)	519 (281)
Long acting β_2_ agonist use (no.)	6 (43%)	8 (53%)
ACQ-6	1.20 (0.72)	1.32 (0.79)
FEV_1_ (litres)	3.57 (3.32–4.03)	3.55 (2.98–4.32)
FEV_1_ (% predicted)	93 (80–98)	94 (78–102)
PC_20_ (mg/mL histamine)	1.30 (0.63–4.73)	1.79 (0.53–3.93)
Fe_NO_ (parts per billion)	30.5 (21.8–67.3)	38.0 (25.3–69.3)
Blood eosinophils (cells × 10^9^/L)	0.30 (0.20–0.40)	0.35 (0.23–0.48)
Total serum IgE (IU/mL)	190 (117–289)	398 (106–692)
Skin prick test responses (total positive)	3.7 (1.5)	4.5 (1.5)
Ex-smoker (no.)	1 (7.1%)	2 (12.5%)
Smoking pack years	0.04 (0.13)	0.53 (2.00)
Ex-smokers only	0.50 (not applicable)	4.25 (5.30)

Data are number (%), mean (SD), or median (IQR).

ACQ, Asthma Control Questionnaire; Fe_NO_, fractional exhaled nitric oxide; FEV_1_, forced expiratory volume in 1 second; IgE, immunoglobulin E; PC_20_, provocation concentration of histamine required to produce a 20% drop in FEV_1_.

### Timapiprant treatment did not affect clinical exacerbation severity

**Figure 2:**
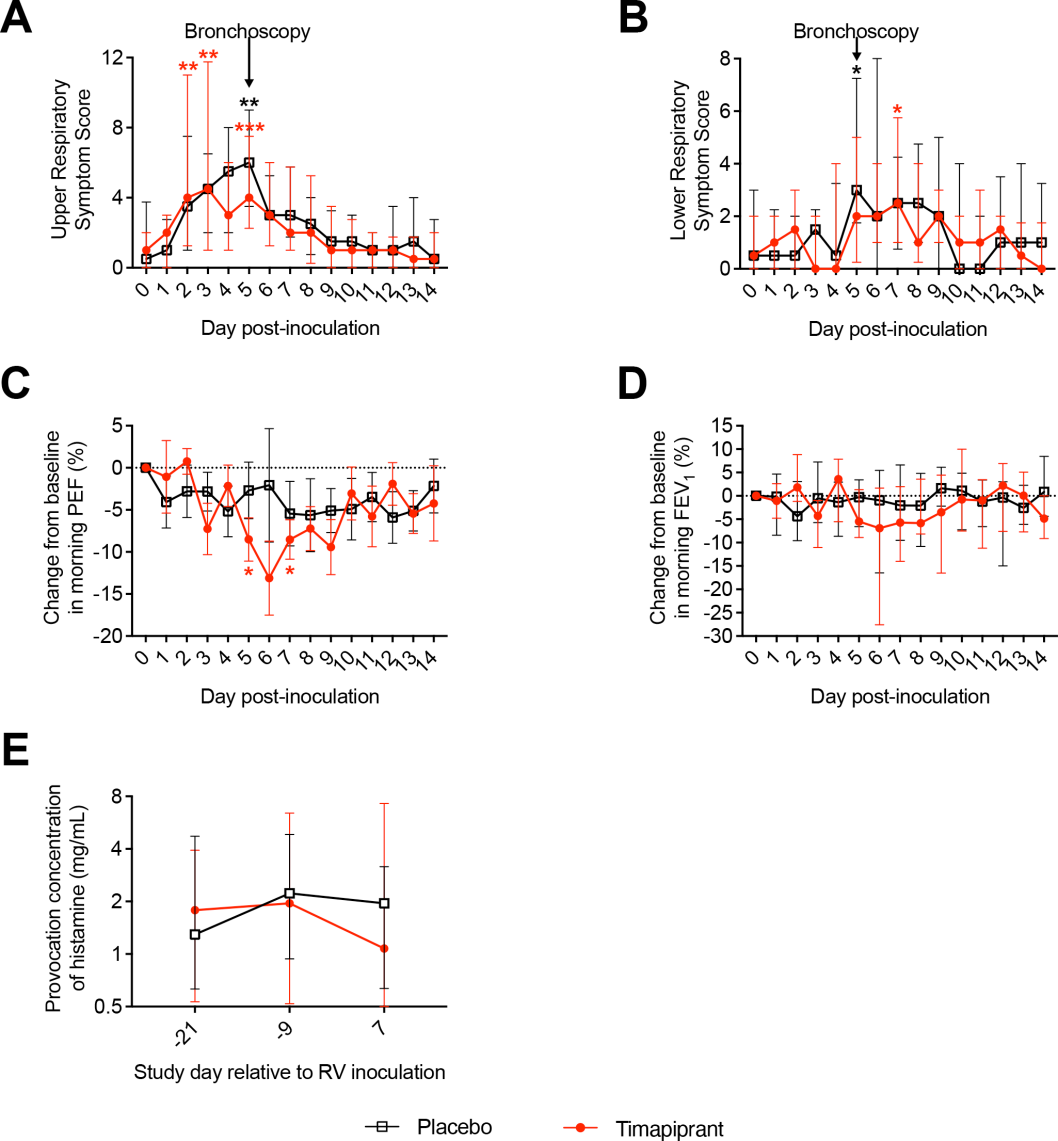
Timapiprant did not alter symptom scores, lung function, or PC_20_ during rhinovirus (RV) infection in subjects with asthma. Thirty patients with asthma were experimentally infected with RV-A16 with n=16 receiving timapiprant and n=14 receiving placebo. (A) Upper and (B) lower respiratory symptom scores during infection. (C) Morning peak expiratory flow (PEF), (D) FEV_1_ and (E) histamine PC_20_ during infection. Values shown are medians and IQR (non-parametric) except (C) morning PEF, shown in mean and SE (parametric). The placebo and timapiprant groups were compared at each time point, with no significant differences, and within each group each time point was compared with baseline (by mixed-effect model with the Geissner-Greenhouse correction followed by Dunnett’s multiple comparison tests for parametric data with missing values, or Friedman test followed by Dunn’s multiple comparisons for non-parametric data). *p<0.05, **p<0.01, ***p<0.001.

Subjects had significantly increased upper and lower respiratory symptoms during RV infection (figure 2A–B). Lung function was reduced during infection compared with baseline, with a statistically significant decline in PEF in the timapiprant group only (figure 2C–D). The median cumulative lower respiratory symptom score in the 14 days post infection was 21.0 (IQR 13.3 to 46.5) in the timapiprant group and 18.0 (9.8 to 51.5) in the placebo group, a difference of 3.0 (95% CI −29.0 to 17.0; p=0.78). There was no difference between the timapiprant-treated and placebo-treated groups in other measures of clinical exacerbation severity, including upper respiratory symptoms, lung function, or airway hyper-responsiveness assessed either individually at each time point or in terms of cumulative area under the curve (AUC) values (figure 2A–E and table 2).

**Table 2:** Lower respiratory symptoms, lung function, Fe_NO_, PC_20_ and ACQ-6

	Placebo	Timapiprant	Difference (timapiprant – placebo) (95% CI)	P value
Total lower respiratory symptom score day 0–14	18.0 (9.8 to 51.5)	21.0 (13.3 to 46.5)	3.0 (−29.0 to 17.0)	0.78
Morning PEF % change from baseline day 0–14 AUC	−41.3 (−145.7 to −2.1)	−73.4 (−171.0 to −40.3)	−32.1 (−102.7 to 77.6)	0.58
Morning FEV_1_ % change from baseline day 0–14 AUC	−13.5 (−93.3 to 17.6)	−45.1 (−106.6 to −3.2)	−31.7 (−84.5 to 59.5)	0.35
Fe_NO_ % change from baseline day 0–10 AUC	−2 (−106 to 264)	294 (−40 to 459)	296 (−98 to 494)	0.28
PC_20_ day 7	1.95 (0.63 to 3.17)	1.08 (0.31 to 7.28)	−0.88 (−2.32 to 4.74)	0.58
ACQ-6 day 10; mean (SD)	1.49 (0.73)	1.32 (0.70)	−0.17 (−0.70 to 0.37)	0.53

Data are median (IQR) except where shown. 95% CIs for median values derived via bootstrapping. AUC scores were derived using the trapezoidal method and are based on AUC over x-axis—AUC under x-axis. Note: one subject in the placebo group was unable to complete PC_20_ on day 7 for logistical reasons and is therefore excluded.

ACQ, Asthma Control Questionnaire; AUC, area under the curve; Fe_NO_, Fractional exhaled nitric oxide; FEV_1_, Forced expiratory volume in 1 second; PC_20_, Provocation concentration of histamine required to produce a 20% drop in FEV_1_; PEF, peak expiratory flow.

### Timapiprant treatment had little effect on virus-induced airway inflammation

**Figure 3:**
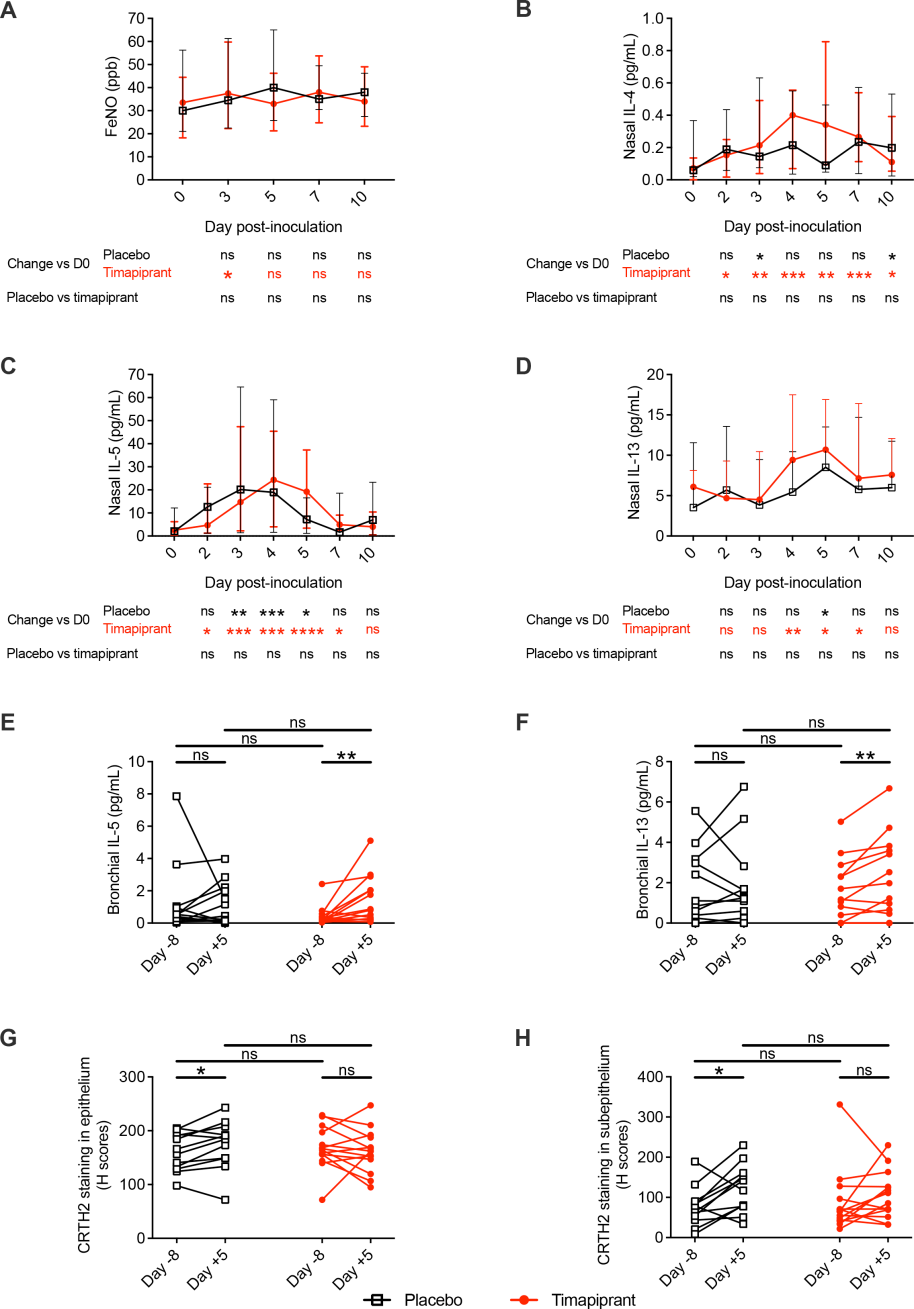
Timapiprant treatment had little effect on virus-induced type 2 airway inflammation. Thirty patients with asthma were experimentally infected with RV-A16 with n=16 receiving timapiprant and n=14 receiving placebo. (A) Fe_NO_ during infection. (B–F) Concentration of interleukin (IL)-4, IL-5 and IL-13 at baseline and during infection in nasosorption (B–D, respectively) and bronchosorption samples (E, F, respectively; IL-4 not shown); there are two fewer bronchosorption samples as two patients did not undergo bronchoscopy for logistical reasons (one in each group). (G, H) CRTH2 staining of epithelial and subepithelial sections, respectively, from before and during infection. Four patients are not included in the biopsy analysis; two who did not undergo bronchoscopy and two who did not have bronchial biopsies on one occasion for technical reasons (one in each group). (A–D) Medians with IQRs. (E–H) Individual subjects. The placebo and timapiprant groups were compared at each time point by Mann-Whitney U tests. Within each group, each time point was compared with baseline by Wilcoxon rank sum tests. *p<0.05, **p<0.01, ***p<0.001 ****p<0.0001.

Having observed no effect on clinical parameters, we next sought to study type 2 and proinflammatory pathways to assess whether timapiprant administration had any effect on RV-induced airway inflammation. RV infection led to statistically significant induction of nasal IL-4, IL-5 and IL-13 in both groups and bronchial IL-5 and IL-13 in the timapiprant group only (figure 3B–F). In keeping with a lack of effect of timapiprant on symptoms, there were no differences between the timapiprant-treated and placebo-treated groups at individual timepoints (figure 3B–F) or in terms of overall AUC for concentrations of the prototypical type 2 cytokines IL-4, IL-5 and IL-13 in nasosorption and bronchosorption samples (online supplemental figure S1; online supplemental table S3) or exhaled breath Fe_NO_, a biomarker of type 2 inflammation (figure 3A; table 2). There was similarly no effect of the drug on the induction of proinflammatory mediators IL-1β, IL-6, IL-8 and tumour necrosis factor, other than a small but statistically significant greater increase in nasal (but not bronchial) IL-1β in the timapiprant group (online supplemental figure S1; online supplemental table S3).

10.1136/thoraxjnl-2021-217429.supp2 Supplementary data

In bronchial biopsy samples, there was evidence of recruitment of CRTH2^+^ cells during RV infection in the placebo group that was absent in the timapiprant-treated subjects, although the difference between the groups was not significant at the 5% level (figure 3G–H). Collectively, these data indicate that type 2 inflammation and proinflammatory responses to virus infection were largely unaffected by timapiprant.

### Timapiprant had no effect on antiviral immune responses

**Figure 4:**
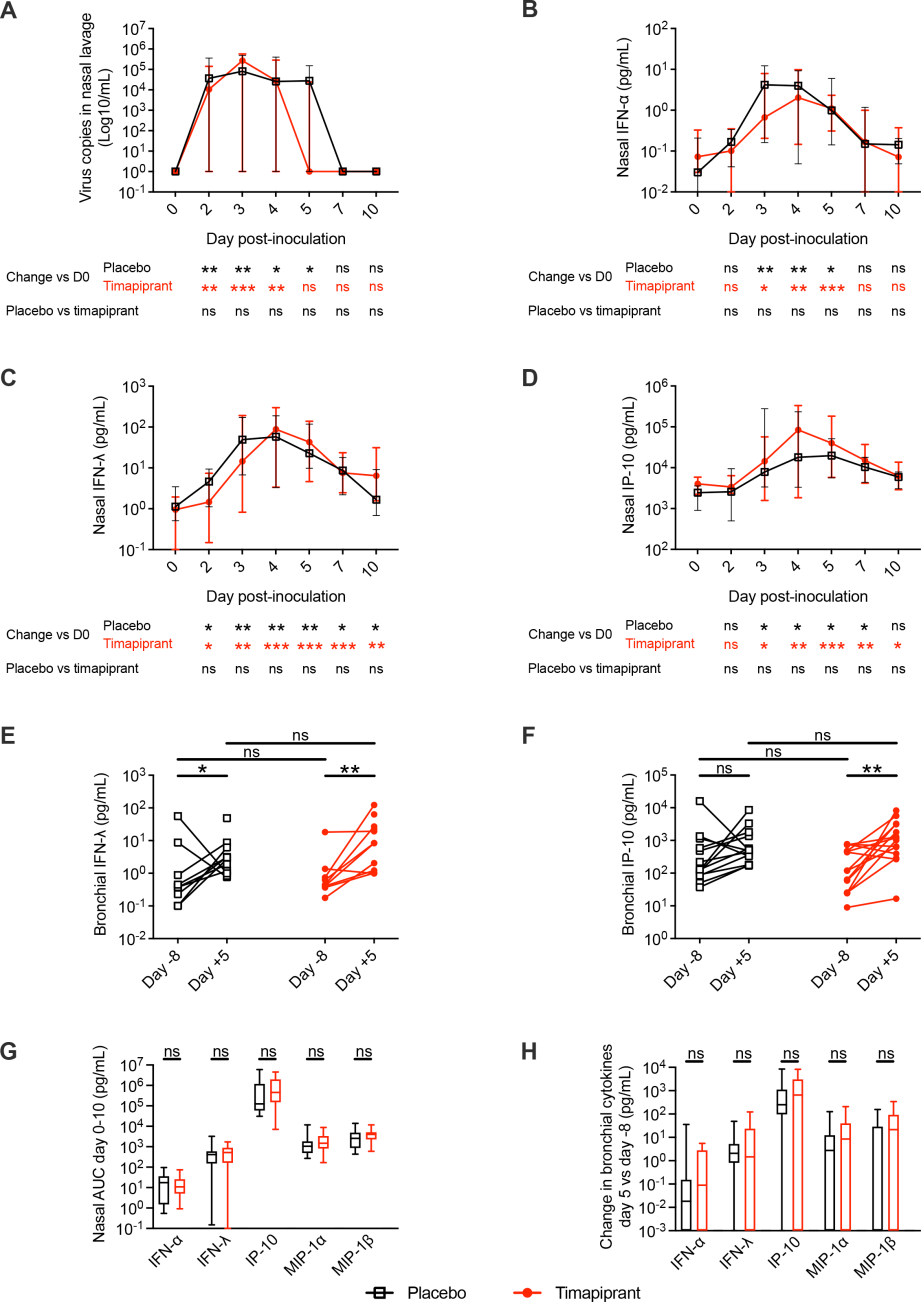
Timapiprant had no effect on virus load or antiviral immune responses. Thirty patients with asthma were experimentally infected with rhinovirus-A16 with n=16 receiving timapiprant and n=14 receiving placebo. (A) Virus copies determined by qPCR expressed as Log_10_ copies per mL of nasal lavage. (B–F) Concentrations of interferon (IFN)-α/-λ and IFN-γ-induced protein (IP)-10 in nasosorption (B–D, respectively) and bronchosorption samples (E, F, respectively; IFN-α not shown) at baseline and during infection. (G) Area under the curve (AUC) during infection of IFN-α/-λ and IFN-induced proteins (IP-10, macrophage inflammatory protein (MIP)1-α, MIP-1β) in nasosorption. (H) Change from baseline to infection in bronchosorption in IFN-α/-λ and IFN-induced proteins. There are two fewer bronchosorption samples as two patients did not undergo bronchoscopy for logistical reasons (one in each group). (A–D) Medians with IQRs. (E, F) Individual subjects. (G, H) Medians (lines) with IQRs (boxes) and full range (whiskers). The placebo and timapiprant groups were compared at each time point by Mann-Whitney U tests. Within each group, each time point was compared with baseline by Wilcoxon rank sum tests. *p<0.05, **p<0.01, ***p<0.001.

Given the lack of anticipated suppression of type 2 inflammation, we next determined whether timapiprant had any effects on antiviral responses and associated virus replication. Virus RNA copies were increased from baseline in both treatment and placebo groups at day 2–4 post inoculation with no effect of timapiprant observed at any time point (figure 4A). RV infection was associated with increased levels of the antiviral mediators interferon (IFN)-α, -λ, and various IFN-inducible proteins (IFN-γ-induced protein (IP)-10 also known as C-X-C motif chemokine ligand (CXCL)10, macrophage inflammatory protein (MIP)1-α and MIP-1β also known as chemokine (C-C motif) ligand (CCL)3 and CCL4, respectively; figure 4B–H; online supplemental table S3). Timapiprant treatment had no effect on levels of IFN-α/-λ at any individual time point (figure 4B–C and E–F) or overall AUC (figure 4G–H; online supplemental table S3) in nasosorption or bronchosorption samples.

Although there were no differences between the groups in virus copies at any time point, virus loads remained significantly elevated at day 5 compared with baseline in the placebo but not the timapiprant group. This could be consistent with faster virus clearance in timapiprant-treated participants, but this is not supported by differences in any other antiviral mediator and could be artefact consequent on small subject numbers.

### PGD_2_ was not induced by RV infection and was unaltered by timapiprant

A previous study from our group demonstrated induction of PGD_2_ following RV challenge in a similar group of subjects with asthma,^25^ providing a rationale for this study. Having failed to observe a convincing effect of CRTH2 antagonism on either symptoms or inflammation, we next investigated whether the endogenous ligand of the target receptor, PGD_2_, had been induced by RV infection in the present study and whether timapiprant altered this. PGD_2_ was readily detectable in nasal and bronchial samples at all timepoints including preinfection, with no induction after RV infection and no effect of timapiprant seen (online supplemental figure S2A–C).

10.1136/thoraxjnl-2021-217429.supp3 Supplementary data

Our previous observation of RV-induced nasal PGD_2_ in asthma^25^ included ICS-naïve (n=11) as well as ICS-treated (n=17) subjects. As the patients in the current study were all on ICS treatment, we postulated that the broad immunosuppressive effects of ICS might mask any potential beneficial effects of concomitant timapiprant. We therefore investigated whether there was a relationship between ICS treatment and nasal PGD_2_ levels and found that nasal PGD_2_ levels were negatively correlated with the prescribed dose of ICS (online supplemental figure S2D). This raises speculation that timapiprant may not have effects on PGD_2_ in addition to those imparted by ICS alone.

### Timapiprant was safe and well-tolerated

The incidence of adverse events was similar in the timapiprant and placebo groups (online supplemental table S4), consistent with previous clinical trials of timapiprant^29 35–39^ and other CRTH2 antagonists. There were no drug-related withdrawals, serious adverse effects or deaths.

## Discussion

This study builds on the literature showing a limited effect of CRTH2 antagonists in asthma^29 30 35 37 40–55^ by assessing their efficacy in the setting of virus-induced acute asthma exacerbation. Despite prior data demonstrating upregulation of the CRTH2-PGD_2_ pathway in asthma^20^ and that targeting the CRTH2 receptor reduces type 2 inflammation in vitro^16 17^ and, to some degree, in vivo (reduction of sputum eosinophils, although no change in blood eosinophils or Fe_NO_),^41^ in the current study the CRTH2 antagonist timapiprant did not attenuate the clinicopathological changes induced by RV infection in asthma. Indeed, the difference between treatment groups in the primary endpoint, median cumulative lower respiratory symptom score in the 14 days post-RV inoculation, was 3.0 (95% CI −29.0 to 17.0; p=0.78).

As in previous RV challenge studies in asthma, RV infection resulted in a worsening of asthma-related inflammation, symptoms and lung function as evidenced by significant increases in type 2 and antiviral inflammatory mediators in bronchial samples (figures 3E–F and 4E–F), in CRTH2^+^ immunostaining in the lower airways (figure 3G–H), Fe_NO_ (day 3, timapiprant-treated group, figure 3A), lower respiratory symptoms (at days 5 and 7 in the placebo-treated and timapiprant-treated groups, respectively; figure 2B), and a significant decline in morning PEF (days 5 and 7, timapiprant-treated group, figure 2C). Timapiprant treatment failed to attenuate this.

We believe it unlikely the lack of effect observed in this study was due to choice of drug, as there is no evidence to suggest timapiprant is less efficacious than any other CRTH2 antagonist. No head-to-head studies have been conducted, but previous comparisons of drug studies have indicated that they are broadly similarly effective.^56^ Timapiprant was selected as a dose-ranging study, not powered to look for a difference in exacerbation rate, reported a significant reduction in respiratory infections (12.3% vs 23.1%; p=0.003) and a non-significant reduction in exacerbations (3.8% vs 7.7%; p=0.107) in the pooled dose group^29^—a signal not seen with any other CRTH2 antagonists. Timapiprant was also the CRTH2 antagonist with the best safety record at the time of study initiation, having been trialled in the largest number of patients including the most with asthma.

The dose administered appeared well justified as the same dose-ranging study of timapiprant found plasma levels well in excess of the equilibrium dissociation constant (K_B_) at the lowest dose, 25 mg once a day, when first measured after 2 weeks treatment. Treatment with 50 mg once a day for 3 weeks prior to RV inoculation, as employed in this study, substantially exceeds this. Moreover, there appeared to be a treatment effect on RV-induced recruitment of CRTH2^+^ cells to the airway wall (recruitment was observed in placebo-treated but not in timapiprant-treated patients), suggesting 3 weeks treatment was sufficient to impact CRTH2^+^ cell recruitment.

There are several explanations for the lack of effect observed in our study. The subjects in this study had moderate rather than severe asthma and the exacerbations were relatively mild, not requiring oral corticosteroids or nebulised bronchodilators. There may therefore have been insufficient inflammation to detect an effect of the drug. In particular, PGD_2_ levels in airway secretions were not increased during infection contrary to the only previous study to measure this.^25^ There were nonetheless readily quantifiable levels of PGD_2_ in the airways to serve as a target for timapiprant. Furthermore, our results are consistent with two recent phase 3 clinical trials that found the CRTH2 antagonist fevipiprant did not reduce the rate of asthma exacerbations requiring systemic corticosteroids in patients with severe asthma, including a subgroup with raised blood eosinophils indicative of ongoing type 2 inflammation.^30^ Our study highlights that controlled experimental RV challenge studies can be used to study the effects of novel therapies using smaller numbers of patients and provide results that are consistent with larger scale trials. The predictive value of this model is currently being directly tested in an ongoing phase 3 clinical trial of timapiprant (EudraCT 2018-003548-22).

It is possible that only a subset of subjects with type 2 asthma benefit from CRTH2 antagonist treatment, and that these were not sufficiently represented in our study to produce a significant result. Indeed, the early trials of anti-IL-5 treatment did not select for patients with raised eosinophils, the cell type regulated by IL-5, and therefore yielded negative results.^57^ However, this study’s cohort resembled a subgroup with the greatest lung function response to timapiprant in an earlier trial: atopic with positive skin prick tests, partially uncontrolled asthma as defined by ACQ-6 score, raised blood eosinophil counts, and relatively young.^29^ No alternative biomarker or clinical parameter for identifying treatment response has been suggested in the literature. The subject numbers in the current study were too small to perform subgroup analyses, but there was no trend to suggest there was a subgroup of responders.

Alternatively, CRTH2 blockade may not offer additional benefits over and above ICS which are potent immunosuppressive agents, as suggested by the observed negative correlation between prescribed ICS dose and PGD_2_ levels during RV infection. Twelve previous studies of CRTH2 antagonists have been conducted in patients taking ICS,^30 41–44 47–50 52 54 55^ only four of which showed any benefit at all with CRTH2 blockade.^30 41–43^ Two of these reported only modest lung function improvements no superior than montelukast (+112 mL and +142 mL greater FEV_1_),^42 43^ while the other two studies found differences in post-bronchodilator but not pre-bronchodilator FEV_1_ and minimal changes in symptom measures.^30 41^ Given that current guidelines recommend all patients with asthma should be on ICS, novel therapies should offer clear additive benefit over and above that conferred by ICS.

Numerous candidates other than PGD_2_ have been proposed as master regulators of type 2 inflammation (eg, IL-25,^58^ IL-33,^5^ thymic stromal lymphopoietin (TSLP)).^59^ PGD_2_-CRTH2 signalling may be redundant in the presence of one or more of these. This would be consistent with the lack of suppression of virus enhancement of type 2 cytokines in the timapiprant group. Of the other candidates, TSLP signalling has been shown to be clinically important as anti-TSLP compounds significantly reduce exacerbations as well as markers of type 2 inflammation (serum IL-5, IL-13, blood eosinophils, Fe_NO_ and total IgE).^60 61^

In conclusion, the CRTH2 antagonist timapiprant did not attenuate RV-induced increases in symptoms and asthma pathophysiology in a group of ICS-treated subjects with partially controlled asthma. The results of the ongoing phase 3 clinical trial of timapiprant in asthma are awaited with interest.

## Data Availability

Data are available upon reasonable request. Anonymised data are available on reasonable request from the corresponding author, Professor Sebastian Johnston.
