# Supplementary material for: Effect of CRTH2 antagonism on the response to experimental rhinovirus infection in asthma: a pilot randomised controlled trial
Source: Thorax. 2021 Oct 29;77(10):950–9. doi: 10.1136/thoraxjnl-2021-217429 (PMC9510426; doi:10.1136/thoraxjnl-2021-217429)
Supplement: Supplementary data [file thoraxjnl-2021-217429supp001.pdf]

BMJ Publishing Group Limited (BMJ) disclaims all liability and responsibility arising from any reliance placed on this supplemental material which has been supplied by the author(s)

Supplementary material

Inclusion and exclusion criteria

Table S1 Inclusion and exclusion criteria

Inclusion criteria	Exclusion criteria
• An Asthma Control Questionnaire (ACQ-6) Score >0.75<br>• Positive histamine challenge test (PC_{20} <8 µg/mL, or <12 µg/mL and bronchodilator response ≥ 12%)<br>• Worsening asthma symptoms with infection since last change in asthma therapy<br>• Positive skin prick test to common aeroallergens (e.g. house dust mite, pollens, animal epithelia)<br>• Treatment comprising inhaled corticosteroids (ICS) or combination inhaler (Long-Acting Beta Agonist with ICS)<br>• Participant is willing for their GP to be informed of their participation<br>• Absence of serum neutralizing antibodies to rhinovirus-A16	• Presence of clinically significant diseases other than asthma (cardiovascular, renal, hepatic, gastrointestinal, haematological, pulmonary, neurological, genitourinary, autoimmune, endocrine, metabolic, neoplasia etc.), which, in the opinion of the investigator, may either put the patient at risk because of participation in the trial, or diseases which may influence the results of the study or the patient's ability to take part in it<br>• Smoking history over past 12 months<br>• Seasonal allergic rhinitis symptoms at screening or during the 3-week pre-treatment phase (prior to rhinovirus inoculation)

Farne H, *et al. Thorax* 2022; 77:950–959. doi: 10.1136/thoraxjnl-2021-217429

	• Asthma exacerbation or viral illness within the previous 6 weeks or during the 3-week pre-treatment phase (prior to rhinovirus inoculation)<br>• Current or concomitant use of oral steroids, anti-leukotrienes or monoclonal antibodies<br>• Pregnant or breast-feeding women. Patients should not be enrolled if they plan to become pregnant during the time of study<br>• Contact with infants <6 months or immunocompromised persons, elderly and infirm at home or at work<br>• Subjects who have known evidence of lack of adherence to medications and/or ability to follow physician's recommendations

Summary of study assessments and sampling

Table S2 Summary of study assessments and sampling

	Screening	*Visit 1 day -21*	*Visit 2 day -9*	*Visit 3 day -8*	*Visit 4 day 0*	*Visit 5 day 2*	*Visit 6 day 3*	*Visit 7 day 4*	*Visit 8 day 5*	*Visit 9 day 7*	*Visit 10 day 10*	*Visit 11 day 42*
ACQ-6	*X*	*X*	*X*		*X*						*X*	*X*
Clinic spirometry	*X*	*X*	*X*		*X*	*X*	*X*	*X*	*X*	*X*	*X*	*X*
Exhaled nitric oxide (FeNO)	*X*	*X*	*X*		*X*		*X*		*X*	*X*	*X*	*X*
Histamine challenge (PC_{20})	*X*	*X*	*X*							*X*		
Blood tests	*X*	*X*		*X*			*X*		*X*		*X*	*X*
*Other screening tests**	*X*											
Home symptom scores		⟶										
Home spirometry		⟶										
Nasosorption and nasal lavage		*X*			*X*	*X*	*X*	*X*	*X*	*X*	*X*	
Bronchoscopy (bronchosorption, biopsies)				*X*					*X*			
Virus inoculation					*X*							

*Other screening tests include: skin prick testing, ECG, urinary pregnancy test, chest radiograph

Symptom scores

Symptoms were assessed by daily diary cards, from randomization three weeks before virus inoculation until six weeks post-inoculation. The daily cold score was measured using the Jackson scale(1) and summated from individual scores (sneezing, headache, malaise, chilliness, nasal discharge, nasal obstruction, sore throat, cough, fever) each graded from 0 (absent) to 3 (severe). The daily lower respiratory score was also summated from individual symptom scores (cough on waking; wheeze on waking; daytime cough; daytime wheeze; daytime chest tightness; daytime shortness of breath; nocturnal cough, wheeze or shortness of breath), also each graded 0–3 (absent, mild, moderate, severe). The same diary cards recorded study medication usage and home spirometry.

Clinical assessments

Spirometry was performed at home using a PiKo-1 (nSpire Health, UK) or Asma-1 handheld spirometer (Vitalograph, UK), and in clinic using a MicroLab spirometer (CareFusion, Kent, UK). FeNO was measured in clinic using a NIOX VERO (Aerocrine AB, Sweden). Bronchoprovocation testing using histamine was undertaken to calculate the provocative concentration of histamine causing a 20% reduction in FEV_1 (PC_{20}) as per previous studies(2, 3).

Sampling procedures

Nasosorption, nasal lavage, and bronchoscopy (including bronchosorption and bronchial biopsies) were all performed as in previous studies(2, 3).

Farne H, *et al. Thorax* 2022; 77:950–959. doi: 10.1136/thoraxjnl-2021-217429

Laboratory methods

Concentrations of soluble mediators were quantified in the eluates from the nasosorption and bronchosorption strips. For PGD_2, a PGD_2-MOX enzyme immunoassay was used (Cayman Chemical Company, USA). The remaining mediators were measured using highly sensitive MesoScale Discovery assays (MesoScale Discovery, USA) as previously described(2), including: IL-1β, IL-4, IL-5, IL-6, IL-8, IL-13, interferon (IFN)-α, IL-29/IFN-λ1, IFN-γ-induced protein (IP)-10 also known as C-X-C motif chemokine ligand (CXCL)10, macrophage inflammatory protein (MIP)1-α and MIP-1β also known as chemokine (C-C motif) ligand (CCL)3 and CCL4 respectively), and tumour necrosis factor (TNF).

Farne H, *et al. Thorax* 2022; 77:950–959. doi: 10.1136/thoraxjnl-2021-217429

BMJ Publishing Group Limited (BMJ) disclaims all liability and responsibility arising from any reliance placed on this supplemental material which has been supplied by the author(s)

Supplementary tables

Table S3 Nasal and bronchial cytokines, CRTH2 staining in bronchial biopsies, and virus copies in nasal lavage

	Placebo	Timapiprant	Difference (timapiprant – placebo) (95% CI)
Nasal IL-4 AUC	2.173 (0.718 to 6.255)	2.234 (1.515 to 4.072)	0.061 (-3.769 to 1.964)
Nasal IL-5 AUC	125.6 (13.73 to 267.1)	91.37 (41.87 to 243.5)	-34.2 (-179.4 to 138.7)
Nasal IL-13 AUC	53.72 (23.24 to 125.9)	82.62 (34.88 to 133.6)	28.9 (-49.2 to 79.5)
Nasal IFN-α AUC	17.54 (1.493 to 36.08)	10.9 (5.076 to 25.58)	-6.64 (-25.55 to 9.28)
Nasal IFN-λ AUC	412.4 (149.9 to 597.5)	518.6 (161.7 to 885.9)	106.2 (-188.4 to 560.1)
Nasal IP-10 AUC	125012 (59252 to 1199856)	454645 (149871 to 1975763)	329634 (-641673 to 1238147)
Nasal MIP-1α AUC	1063 (491.2 to 1841)	1476 (808.5 to 3350)	413.4 (-519.0 to 1689.2)
Nasal MIP-1β AUC	2556 (865.2 to 4913)	4025 (2267 to 5143)	1469 (-1098.5 to 3018.7)

Nasal IL-1β AUC	675.1 (477.8 to 1449)	1704 (635.2 to 3153)	1029 (-284.0 to 2124.5)
Nasal IL-6 AUC	606.9 (152.2 to 2187)	677.6 (250 to 1379)	70.75 (-1331 to 749.4)
Nasal IL-8 AUC	54661 (40977 to 79169)	72289 (39676 to 193940)	17628 (-18841 to 120489)
Nasal TNF AUC	40.79 (21.5 to 88.69)	54.04 (28.18 to 110.8)	13.25 (-41.52 to 64.38)
Nasal PGD2 AUC	9.167 (7.415 to 12.83)	8.355 (6.67 to 10.86)	-0.812 (-4.707 to 1.699)
Bronchial IL-4 (change D+5 vs D-8)	0 (-0.01643 to 0.02396)	0.001883 (-0.01107 to 0.02885)	0.002 (-0.010 to 0.031)
Bronchial IL-5 (change D+5 vs D-8)	0.04902 (-0.3711 to 1.149)	0.5004 (-0.0003907 to 1.751)	0.451 (-0.706 to 1.792)
Bronchial IL-13 (change D+5 vs D-8)	0 (-0.7222 to 0.7579)	0.313 (0 to 1.282)	0.313 (-0.385 to 1.276)
Bronchial IFN-α (change D+5 vs D-8)	0 (-0.4273 to 0.1341)	0.08973 (-0.1404 to 2.786)	0.090 (-0.153 to 2.518)
Bronchial IFN-λ (change D+5 vs D-8)	1.521 (0.3973 to 4.482)	1.463 (0 to 23.69)	-0.058 (-2.621 to 15.732)

Farne H, *et al. Thorax* 2022; 77:950–959. doi: 10.1136/thoraxjnl-2021-217429

Bronchial IP-10 (change D+5 vs D-8)	226.7 (-280.6 to 1033)	646.9 (-23.64 to 3042)	420.2 (-487.6 to 2226.0)
Bronchial MIP-1α (change D+5 vs D-8)	2.092 (-18.2 to 12.31)	8.443 (-7.48 to 39.91)	6.351 (-12.564 to 34.852)
Bronchial MIP-1β (change D+5 vs D-8)	-6.86 (-46.08 to 24.83)	21.33 (-5.472 to 92.67)	28.188 (-16.34 to 83.00)
Bronchial IL-1β (change D+5 vs D-8)	-0.4316 (-16.37 to 6.322)	3.236 (-2.482 to 13.79)	3.668 (-4.223 to 19.461)
Bronchial IL-6 (change D+5 vs D-8)	0.9457 (-9.82 to 4.205)	2.542 (-0.8302 to 8.526)	1.596 (-2.976 to 11.159)
Bronchial IL-8 (change D+5 vs D-8)	93.01 (-851.4 to 285.7)	182.7 (-84.81 to 507.1)	89.66 (-240.53 to 1032.24)
Bronchial TNF (change D+5 vs D-8)	0.09044 (-0.5547 to 0.3608)	0.1421 (0.01724 to 0.5016)	0.053 (-0.189 to 0.569)
Bronchial PGD2 (change D+5 vs D-8)	-0.00336 (-0.1476 to 0.1735)	-0.1542 (-0.3205 to 0.1722)	-0.151 (-0.433 to 0.194)
CRTH2 staining in epithelium	16.35 (-9.925 to 29.57)	-11.64 (-41.48 to 23.72)	-27.99 (-61.08 to 18.08)
CRTH2 staining in subepithelium	63.4 (-18.78 to 87.62)	18.21 (-11.82 to 63.75)	-45.19 (-82.30 to 38.31)
Virus copies in nasal lavage (AUC)	479828 (31650 to 5859697)	708923 (100546 to 3623753)	229095 (-2873623 to 2142014)

Farne H, et al. Thorax 2022; 77:950–959. doi: 10.1136/thoraxjnl-2021-217429

Data are median (IQR). 95% confidence intervals (CI's) for median values derived via bootstrapping. AUC scores were derived using the trapezoidal method and are based on AUC over x-axis – AUC under x-axis. Note that two subjects did not undergo bronchoscopy for logistical reasons, one from each group, and the baseline bronchosorption sample for one further subject was lost and therefore that subject is also excluded from the bronchial cytokine data above (timapiprant arm).

Farne H, *et al. Thorax* 2022; 77:950–959. doi: 10.1136/thoraxjnl-2021-217429

Table S4 Adverse events in the safety set

	Placebo (n=22)	Timapiprant (n=22)
Adverse events (number)		
Mild	10	17
Moderate	5	0
Adverse events (type)		
Headache	5	17
Musculoskeletal pain	2	0
Pneumonia	1	0
Respiratory tract infection	1	0
Fever post-bronchoscopy	1	0
Cough	1	0
Sore throat	1	0
Periodontitis	1	0
Periorbital oedema	1	0
Fractured clavicle	1	0
Subjects with adverse events	9/22 (40.9%)	5/22 (22.7%)

Farne H, *et al. Thorax* 2022; 77:950–959. doi: 10.1136/thoraxjnl-2021-217429

Supplementary figure legends

Figure S1 Timapiprant treatment had no effect on virus-induced non-type 2 airway inflammation. 30 patients with asthma were experimentally infected with RV-A16 with n=16 receiving timapiprant and n=14 receiving placebo. (A-D) and (F-I) Concentration of IL-1β, IL-6, IL-8 and TNF at baseline and during infection in nasosorption (A-D respectively) and bronchosorption samples (F-I respectively). There are two fewer bronchosorption samples as two patients did not undergo bronchoscopy for logistical reasons (one in each group). (E) AUC during infection of type 2 (IL-4, IL-5, IL-13) and proinflammatory cytokines (IL-1β, IL-6, IL-8, TNF) in nasosorption. (I) Change from baseline to infection in bronchosorption in type 2 and proinflammatory cytokines. (A-D) and (F-I) show medians with interquartile ranges, (E) and (J) show medians (lines) with interquartile ranges (boxes) and full range (whiskers). The placebo and timapiprant groups were compared at each time point by Mann-Whitney U tests. Within each group, each time point was compared to baseline by Wilcoxon rank sum tests. *$P < 0.05$ **$P < 0.01$ ***$P < 0.001$.

Figure S2 PGD_2 levels in the airways were not affected by RV infection or timapiprant treatment. 30 patients with asthma were experimentally infected with RV-A16 with n=16 receiving timapiprant and n=14 receiving placebo. (A-C) Concentrations of PGD_2 in nasosorption (A) and bronchosorption samples (B) at baseline and during infection, and in terms of AUC for nasosorption (C). There are two fewer bronchosorption samples as two patients did not undergo bronchoscopy for logistical reasons (one in each group). (D) Correlation between prescribed ICS dose and nasal PGD_2 AUC during infection. (A) and (C) show medians with interquartile ranges. The placebo and timapiprant groups were compared by Mann-Whitney tests. Within each group, each time point was compared to baseline by Wilcoxon rank sum

Farne H, *et al. Thorax* 2022; 77:950–959. doi: 10.1136/thoraxjnl-2021-217429

tests. The relationship between prescribed ICS dose and nasal PGD_2 AUC was analyzed by linear regression.

Farne H, *et al. Thorax* 2022; 77:950–959. doi: 10.1136/thoraxjnl-2021-217429

References

1. Jackson GG, Dowling HF, Spiesman IG, Boand AV. Transmission of the common cold to volunteers under controlled conditions. I. The common cold as a clinical entity. AMA archives of internal medicine. 1958;101(2):267-78.

2. Jackson DJ, Makrinioti H, Rana BM, Shamji BW, Trujillo-Torralbo MB, Footitt J, et al. IL-33-dependent type 2 inflammation during rhinovirus-induced asthma exacerbations in vivo. American journal of respiratory and critical care medicine. 2014;190(12):1373-82.

3. Message SD, Laza-Stanca V, Mallia P, Parker HL, Zhu J, Kebadze T, et al. Rhinovirus-induced lower respiratory illness is increased in asthma and related to virus load and Th1/2 cytokine and IL-10 production. Proceedings of the National Academy of Sciences of the United States of America. 2008;105(36):13562-7.

Farne H, *et al. Thorax* 2022; 77:950–959. doi: 10.1136/thoraxjnl-2021-217429
