# Supplementary figures and images for: Effect of CRTH2 antagonism on the response to experimental rhinovirus infection in asthma: a pilot randomised controlled trial
Source: Thorax. 2021 Oct 29;77(10):950–9. doi: 10.1136/thoraxjnl-2021-217429 (PMC9510426; doi:10.1136/thoraxjnl-2021-217429)

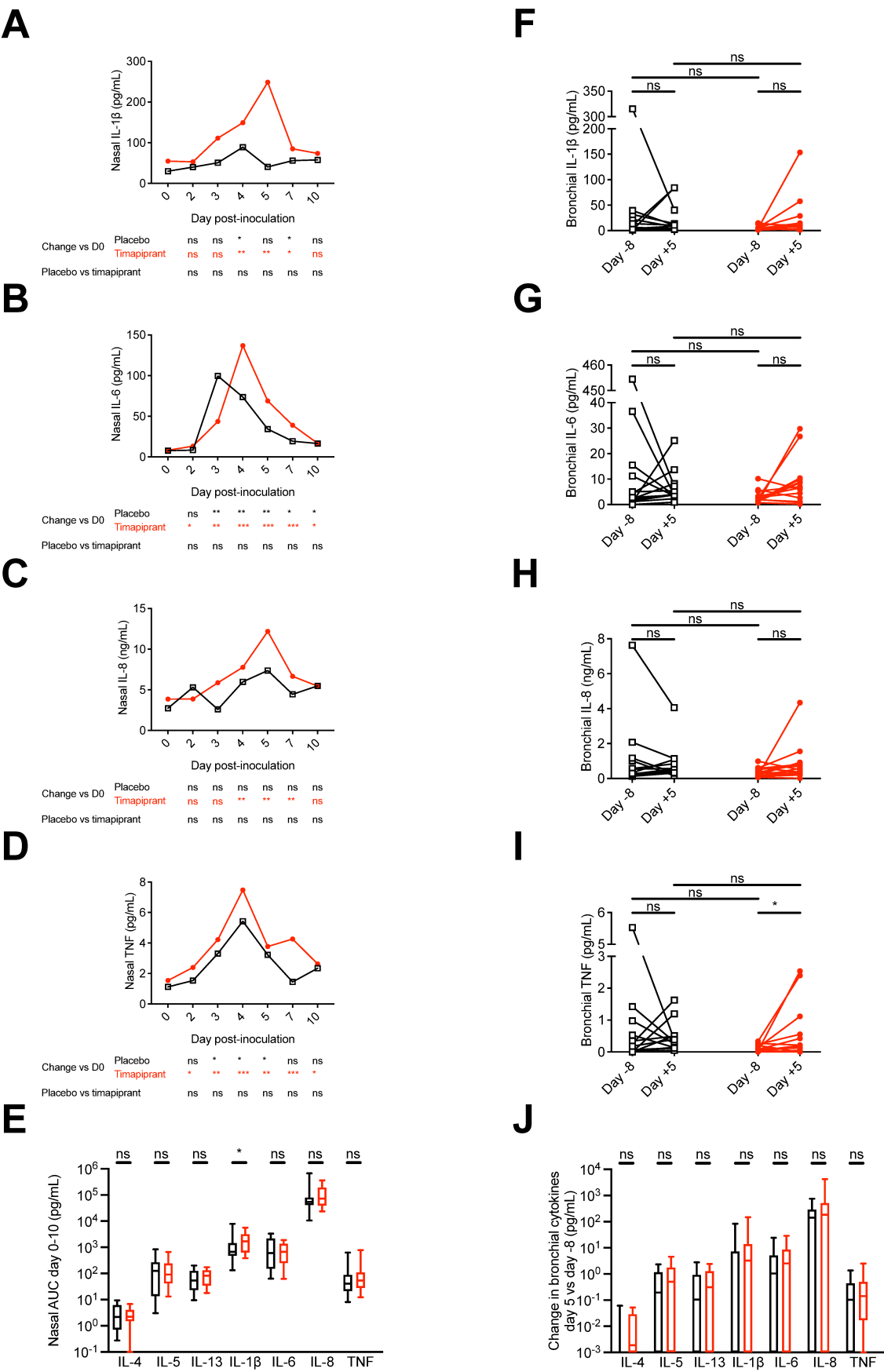

Supplement: Supplementary data [file thoraxjnl-2021-217429supp002.pdf]

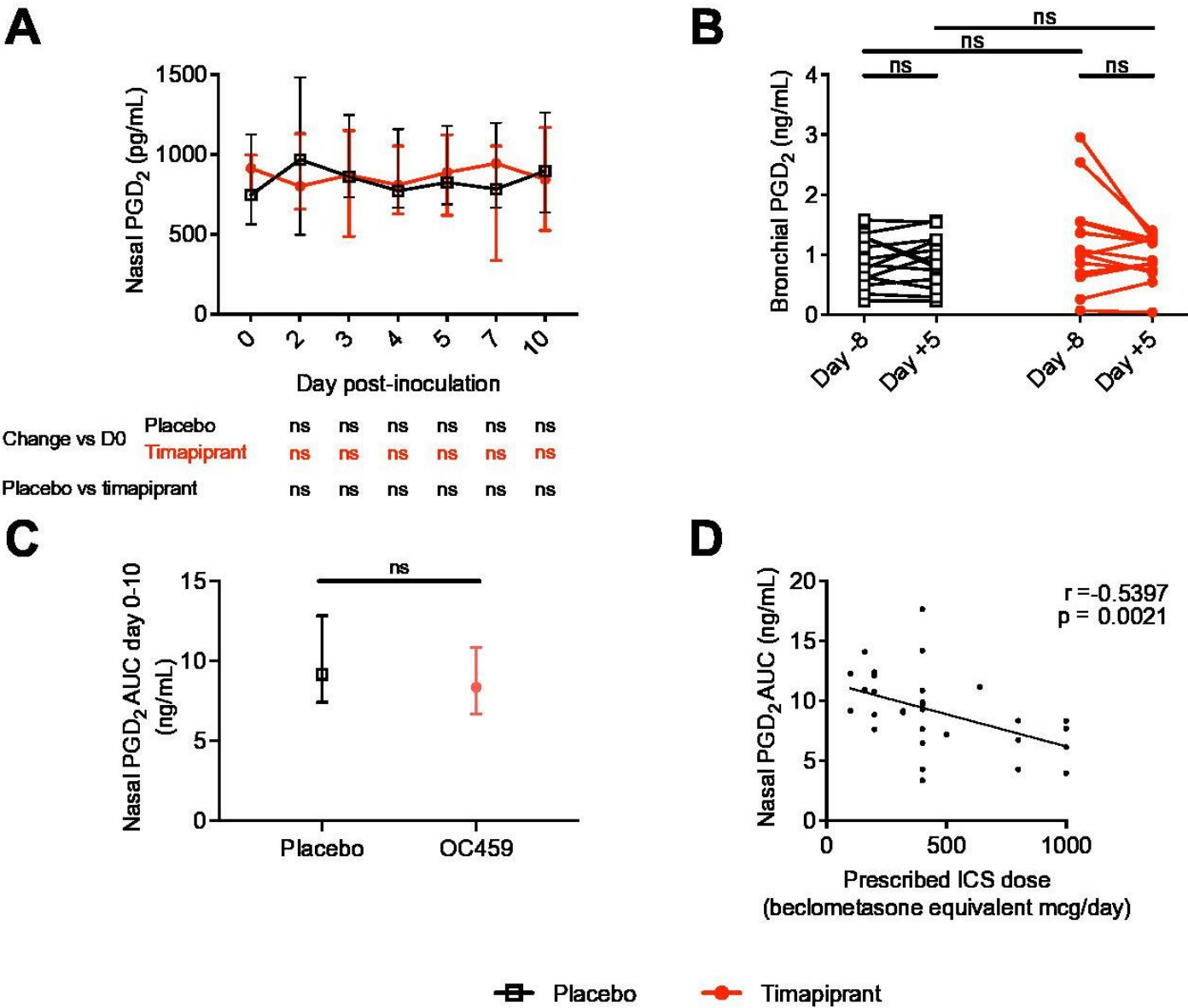

Supplement: Supplementary data [file thoraxjnl-2021-217429supp003.pdf]
